# Supplementary material for: Anticholinergic burden: First comprehensive analysis using claims data shows large variation by age and sex
Source: PLoS One. 2021 Jun 30;16(6):e0253336. doi: 10.1371/journal.pone.0253336 (PMC8244868; doi:10.1371/journal.pone.0253336)
Supplement: S1 Table — (DOCX) [file pone.0253336.s001.docx]

**S1 Table. Description of study population stratified by anticholinergic burden measured through anticholinergic cognitive burden (ACB) score**

|  | **ACB score^a^** | | | | |
| --- | --- | --- | --- | --- | --- |
|  | **ACB = 0 N = 10,679,265** | **ACB = 1 N = 3,084,567** | **ACB = 2 N = 1,236,096** | **ACB ≥ 3 N = 1,471,018** | |
| **Indicators of health care utilization** |  |  |  |  | |
| % hospitalized time [mean (std)] | 0.2 (1.9) | 0.7 (3.5) | 1.4 (5.0) | 2.5 (6.9) | |
| Number of hospital visits (last 12 months) [mean (std)] | 0.1 (0.5) | 0.3 (0.8) | 0.4 (1.0) | 0.6 (1.3) | |
| Number of different prescribers (last 12 months) [mean (std)] | 1.2 (1.2) | 2.1 (1.5) | 2.6 (1.7) | 3.2 (2.0) | |
| Nursing home residence (N, %) | 758,821 (7.1%) | 233,924 (7.6%) | 116,659 (9.4%) | 213,895 (14.5%) | |
| **Morbidities**^b^ |  |  |  |  | |
| Hepatic diseases | 1,207,053 (11.3%) | 695,821 (22.6%) | 369,958 (29.9%) | 496,818 (33.8%) | |
| Renal diseases | 483,697 (4.5%) | 330,562 (10.7%) | 200,061 (16.2%) | 305,793 (20.8%) | |
| Urinary incontinence/overactive bladder | 1,345,519 (12.6%) | 695,486 (22.5%) | 363,664 (29.4%) | 580,722 (39.5%) | |
| Musculoskeletal diseases | 2,353,398 (22.0%) | 1,309,147 (42.4%) | 680,333 (55.0%) | 894,891 (60.8%) | |
| Fractures | 1,531,502 (14.3%) | 563,004 (18.3%) | 273,283 (22.1%) | 380,017 (25.8%) | |
| Neurological diseases | 85,384 (0.8%) | 92,043 (3.0%) | 76,698 (6.2%) | 166,833 (11.3%) | |
| Endocrine and metabolic diseases | 1,607,938 (15.1%) | 976,001 (31.6%) | 512,543 (41.5%) | 659,923 (44.9%) | |
| Psychiatric and behavioral diseases | 2,566,724 (24.0%) | 1,228,545 (39.8%) | 642,079 (51.9%) | 930,621 (63.3%) | |
| Malignancies^c^ | 666,857 (6.2%) | 381,763 (12.4%) | 210,275 (17.0%) | 310,101 (21.1%) | |
| Pain^d^ | 4,865,430 (45.6%) | 1,912,384 (62.0%) | 871,759 (70.5%) | 1,072,768 (72.9%) | |
| Allergy and allergic reactions | 5,290,145 (49.5%) | 1,849,125 (59.9%) | 773,296 (62.6%) | 960,582 (65.3%) | |
| Glaucoma | 612,534 (5.7%) | 378,308 (12.3%) | 211,231 (17.1%) | 281,685 (19.1%) | |
| Pressure ulcer | 35,595 (0.3%) | 37,203 (1.2%) | 30,634 (2.5%) | 64,109 (4.4%) | |
| Obesity^e^ | 1,300,361 (12.2%) | 713,367 (23.1%) | 366,621 (29.7%) | 481,466 (32.7%) | |
| Dizziness | 1,284,647 (12.0%) | 650,739 (21.1%) | 333,482 (27.0%) | 473,493 (32.2%) | |
| Drug abuse^e^ | 108,796 (1.0%) | 53,822 (1.7%) | 36,987 (3.0%) | 87,495 (5.9%) | |
| Alcohol abuse^e^ | 269,446 (2.5%) | 126,786 (4.1%) | 71,579 (5.8%) | 114,332 (7.8%) | |
| Smoking^e^ | 796,549 (7.5%) | 366,466 (11.9%) | 181,448 (14.7%) | 240,037 (16.3%) | |
| **Medication excluding MAC**^f^ |  |  |  |  | |
| Cardiovascular therapy | 1,609,954 (15.1%) | 1,271,979 (41.2%) | 667,152 (54.0%) | 868,316 (59.0%) | |
| Analgesics | 2,838,255 (26.6%) | 1,278,585 (41.5%) | 587,424 (47.5%) | 872,853 (59.3%) | |
| Anti-Parkinson medication | 9,477 (0.1%) | 25,340 (0.8%) | 27,786 (2.2%) | 86,378 (5.9%) | |
| Anti-diabetic medication | 100,328 (0.9%) | 270,968 (8.8%) | 187,781 (15.2%) | 228,417 (15.5%) | |
| Glucocorticoids | 467,852 (4.4%) | 360,975 (11.7%) | 197,063 (15.9%) | 290,675 (19.8%) | |
| Medication for rheumatic diseases | 151,154 (1.4%) | 177,267 (5.7%) | 126,233 (10.2%) | 165,591 (11.3%) | |
| Medication for sleep disorders | 52,469 (0.5%) | 59,755 (1.9%) | 49,580 (4.0%) | 117,386 (8.0%) | |
| Muscle relaxants | 105,237 (1.0%) | 70,818 (2.3%) | 36,823 (3.0%) | 70,627 (4.8%) | |
| Psychiatric medication | 279,673 (2.6%) | 370,697 (12.0%) | 298,413 (24.1%) | 647,249 (44.0%) | |
| COPD-Treatment | 706,344 (6.6%) | 400,004 (13.0%) | 191,302 (15.5%) | 289,864 (19.7%) | |
| Anti-neoplastic treatment | 13,015 (0.1%) | 12,278 (0.4%) | 9,531 (0.8%) | 20,916 (1.4%) | |
| ^a^ Categorization based on the highest level of the ACB score ever reached during the study period. | | | | |  |
| ^b^ Assessed any time prior to start of study period | | | | |  |
| ^c^ Excluding non-melanoma skin malignancies | | | | |  |
| ^d^ Sensitive definition including a wide variety of ICD-10-GM diagnoses related to pain. | | | | |  |
| ^e^ Based on proxy ICD-10-GM codes related to the respective condition.  ^f^ Assessed within 365 days before start of study period (excluding start of study period) | | | | |  |
