# Supplementary material for: Anticholinergic burden: First comprehensive analysis using claims data shows large variation by age and sex
Source: PLoS One. 2021 Jun 30;16(6):e0253336. doi: 10.1371/journal.pone.0253336 (PMC8244868; doi:10.1371/journal.pone.0253336)
Supplement: Attachment — Submitted filename: Reinold_et_al_2021_Response to Reviewers.docx [file pone.0253336.s002.docx]

Please include the following items when submitting your revised manuscript:

- A rebuttal letter that responds to each point raised by the academic editor and reviewer(s). You should upload this letter as a separate file labeled 'Response to Reviewers'.
- A marked-up copy of your manuscript that highlights changes made to the original version. You should upload this as a separate file labeled 'Revised Manuscript with Track Changes'.
- An unmarked version of your revised paper without tracked changes. You should upload this as a separate file labeled 'Manuscript'.

If you would like to make changes to your financial disclosure, please include your updated statement in your cover letter. Guidelines for resubmitting your figure files are available below the reviewer comments at the end of this letter.

If applicable, we recommend that you deposit your laboratory protocols in protocols.io to enhance the reproducibility of your results. Protocols.io assigns your protocol its own identifier (DOI) so that it can be cited independently in the future. For instructions see: <http://journals.plos.org/plosone/s/submission-guidelines#loc-laboratory-protocols>

We look forward to receiving your revised manuscript.

Kind regards,

Andrea Gruneir
Academic Editor
PLOS ONE

Journal Requirements:

When submitting your revision, we need you to address these additional requirements.

1. Please ensure that your manuscript meets PLOS ONE's style requirements, including those for file naming. The PLOS ONE style templates can be found at

<https://journals.plos.org/plosone/s/file?id=wjVg/PLOSOne_formatting_sample_main_body.pdf> and

<https://journals.plos.org/plosone/s/file?id=ba62/PLOSOne_formatting_sample_title_authors_affiliations.pdf>

2.  We note that the grant information you provided in the ‘Funding Information’ and ‘Financial Disclosure’ sections do not match.

We thank the editor for this remark. We changed the funding information from “This study was funded entirely by internal funds of the Leibniz Institute for Prevention Research and Epidemiology – BIPS. The funding institution had no influence on any part of this article.” to *“The author(s) received no external funding for this work.”*

When you resubmit, please ensure that you provide the correct grant numbers for the awards you received for your study in the ‘Funding Information’ section.

3. Thank you for stating the following in the Competing Interests section:

"UH, OR, MB and JR are working at an independent, non-profit research institute, the Leibniz Institute for Prevention Research and Epidemiology – BIPS. Unrelated to this study, BIPS occasionally conducts studies financed by the pharmaceutical industry. Almost exclusively, these are post-authorization safety studies (PASS) requested by health authorities. The design and conduct of these studies as well as the interpretation and publication are not influenced by the pharmaceutical industry. The study presented was not funded by the pharmaceutical industry. The authors have no relevant financial or non-financial interests to disclose."

Please confirm that this does not alter your adherence to all PLOS ONE policies on sharing data and materials, by including the following statement: "This does not alter our adherence to  PLOS ONE policies on sharing data and materials.” (as detailed online in our guide for authors http://journals.plos.org/plosone/s/competing-interests).  If there are restrictions on sharing of data and/or materials, please state these. Please note that we cannot proceed with consideration of your article until this information has been declared.

🡪 We thank the editor for this suggestion. We have added the suggested phrase to the COI statement *“Moreover, this does not alter our adherence to PLOS ONE policies on sharing data and materials.”*

Please include your updated Competing Interests statement in your cover letter; we will change the online submission form on your behalf.

Please know it is PLOS ONE policy for corresponding authors to declare, on behalf of all authors, all potential competing interests for the purposes of transparency. PLOS defines a competing interest as anything that interferes with, or could reasonably be perceived as interfering with, the full and objective presentation, peer review, editorial decision-making, or publication of research or non-research articles submitted to one of the journals. Competing interests can be financial or non-financial, professional, or personal. Competing interests can arise in relationship to an organization or another person. Please follow this link to our website for more details on competing interests: http://journals.plos.org/plosone/s/competing-interests

4. We note that you have indicated that data from this study are available upon request. PLOS only allows data to be available upon request if there are legal or ethical restrictions on sharing data publicly. For information on unacceptable data access restrictions, please see <http://journals.plos.org/plosone/s/data-availability#loc-unacceptable-data-access-restrictions>.

In your revised cover letter, please address the following prompts:

a) If there are ethical or legal restrictions on sharing a de-identified data set, please explain them in detail (e.g., data contain potentially identifying or sensitive patient information) and who has imposed them (e.g., an ethics committee). Please also provide contact information for a data access committee, ethics committee, or other institutional body to which data requests may be sent.

🡪 We thank the editor for this suggestion. We modified the section “Availability of data and material” as follows and included it in the updated version of the cover letter.

*In Germany, use of personal data is protected by the Federal Data Protection Act and particularly the use of claims data for research is regulated by the Code of Social Law. Researchers have to apply for a project-specific permit from the statutory health insurance providers which then need an approval from their governing authorities. The use of the data on which this publication is based was only allowed for BIPS employees within the framework of the specified project and limited to a pre-defined time span. Researchers who want to access the data on which this publication is based need to ask for new approval by the statutory health insurance providers DAK-Gesundheit (*[*service@dak.de*](mailto:service@dak.de)*), die Techniker (*[*service@tk.de*](mailto:service@tk.de)*), hkk Krankenkasse (*[*info@hkk.de*](mailto:info@hkk.de)*) and AOK Bremen/Bremerhaven (*[*info@hb.aok.de*](mailto:info@hb.aok.de)*) which upon granting approval would have to ask their respective authorities for approval. Please contact* [*gepard@leibniz-bips.de*](mailto:gepard@leibniz-bips.de) *for help with this process. The authors confirm that they had no special access privileges to the data and that other researchers will be able to access the data in the same manner as the authors by following the instructions described above.*

b) If there are no restrictions, please upload the minimal anonymized data set necessary to replicate your study findings as either Supporting Information files or to a stable, public repository and provide us with the relevant URLs, DOIs, or accession numbers. Please see <http://www.bmj.com/content/340/bmj.c181.long> for guidelines on how to de-identify and prepare clinical data for publication. For a list of acceptable repositories, please see <http://journals.plos.org/plosone/s/data-availability#loc-recommended-repositories>.

We will update your Data Availability statement on your behalf to reflect the information you provide.

5. We note that you have included the phrase “data not shown” in your manuscript. Unfortunately, this does not meet our data sharing requirements. PLOS does not permit references to inaccessible data. We require that authors provide all relevant data within the paper, Supporting Information files, or in an acceptable, public repository. Please add a citation to support this phrase or upload the data that corresponds with these findings to a stable repository (such as Figshare or Dryad) and provide and URLs, DOIs, or accession numbers that may be used to access these data. Or, if the data are not a core part of the research being presented in your study, we ask that you remove the phrase that refers to these data.

🡪 We thank the editor for pointing this out. The paragraph in question has been rewritten and the phrase “data not shown” is no longer in the manuscript.

6. Your ethics statement should only appear in the Methods section of your manuscript. If your ethics statement is written in any section besides the Methods, please move it to the Methods section and delete it from any other section. Please ensure that your ethics statement is included in your manuscript, as the ethics statement entered into the online submission form will not be published alongside your manuscript.

🡪 We thank the editor for this remark. We have moved the ethics statement to the methods section (Page 4, Line 109-115)

[Note: HTML markup is below. Please do not edit.]

Reviewers' comments:

Reviewer's Responses to Questions

**Comments to the Author**

1. Is the manuscript technically sound, and do the data support the conclusions?

The manuscript must describe a technically sound piece of scientific research with data that supports the conclusions. Experiments must have been conducted rigorously, with appropriate controls, replication, and sample sizes. The conclusions must be drawn appropriately based on the data presented.

Reviewer #1: Yes

Reviewer #2: Partly

2. Has the statistical analysis been performed appropriately and rigorously?

Reviewer #1: Yes

Reviewer #2: Yes

3. Have the authors made all data underlying the findings in their manuscript fully available?

The [PLOS Data policy](http://www.plosone.org/static/policies.action#sharing) requires authors to make all data underlying the findings described in their manuscript fully available without restriction, with rare exception (please refer to the Data Availability Statement in the manuscript PDF file). The data should be provided as part of the manuscript or its supporting information, or deposited to a public repository. For example, in addition to summary statistics, the data points behind means, medians and variance measures should be available. If there are restrictions on publicly sharing data—e.g. participant privacy or use of data from a third party—those must be specified.

Reviewer #1: No

Reviewer #2: Yes

4. Is the manuscript presented in an intelligible fashion and written in standard English?

PLOS ONE does not copyedit accepted manuscripts, so the language in submitted articles must be clear, correct, and unambiguous. Any typographical or grammatical errors should be corrected at revision, so please note any specific errors here.

Reviewer #1: Yes

Reviewer #2: Yes

5. Review Comments to the Author (Please note that all references to pages and lines refer to the clean version of the manuscript)

Please use the space provided to explain your answers to the questions above. You may also include additional comments for the author, including concerns about dual publication, research ethics, or publication ethics. (Please upload your review as an attachment if it exceeds 20,000 characters)

Reviewer #1: The authors present a descriptive cohort study to describe the anticholinergic burden among a general population from German. The authors found identified that anticholinergic burden (ACB) increased in females and older patients, which is well known from other studies. However, the main contribution to the literature are the findings in younger populations, which are often excluded in studies of ACB. Additionally, the analyses stratifying by medication class and prescriber are interesting, and can be important to help identify target groups for interventions to minimize ACB in practice.


Major comments
1. In the write-up of the study design and study population, it would be helpful if the authors specified the study design used as per the STROBE guidelines.

We thank the reviewer for this remark. We now specify the study design in the section “study design and study population” (page 2, line 55):

*Page 2, line 55: We conducted a cross-sectional study using data from the year 2016, the most recent data at the time of analysis.*

2. It may also be helpful if the authors were to provide a graphical depiction of the study design, particularly relating to the inclusion and exclusion criteria (For examples, see Schneeweiss S. et al. Ann Int Med 2019. PMID: 30856654)

🡪 We thank the reviewer for this suggestion. We have created a graphical depiction of the study design (Figure 1). The example by Schneeweiss et al. is very clear and nicely illustrates the inclusion and exclusion criteria. As the example by Schneeweis et al. refers to a longitudinal study design whereas our study has a cross-sectional design, we adapted the template to make sure that there is no misunderstanding regarding the study design, while making sure that the inclusion and exclusion criteria are clearly depicted.

3. A study flow diagram of patient inclusion would be useful (as per STROBE guidelines).

🡪 We have followed the reviewer’s advice and have created a flow diagram to visualize the inclusion and exclusion process (Figure 2).

4. There seems to be a disconnect between the description in text and choice for supplementary vs. in-text tables. For example, the information presented in the appendix table contributes to a substantial portion of the text, and even one of the main conclusions in the first paragraph of the discussion. Conversely, very little attention is paid to Table 1 in the manuscript. I would consider to switch these tables, or consider combining both in one larger table?
5. Similar to the above, perhaps the authors clarify the purpose of Table 1?

🡪 We agree with the reviewer’s remarks. We have switched tables T1 and S1. The purpose of S1 (formerly T1) was to generally describe patients regarding health care utilization, morbidities and medication stratified by anticholinergic burden. As expected, patients with higher anticholinergic burden more often had chronic disease and more often received various types of pharmacological therapy.

6. How the medications were identified in Table 2? Here, I believe these are only the medications that were dispensed in the observation period in 2016? This does or does not include prescriptions dispensed prior to baseline? Perhaps a study design diagram could help clarify this, as recommended above.

🡪 We thank the reviewer for this comment. Table 2 does indeed contain only medications that were dispensed during the study period in 2016. In order to make this clearer we have followed the reviewer’s advice and created a graphical depiction of the study design (new Figure 1) and now also stress it in the methods section. Moreover, we have modified the title of Table 2 to “Prevalence of use of medications with anticholinergic activity (MACs) in persons with anticholinergic burden measured through the anticholinergic cognitive burden (ACB) scale during the observation period (2016)”

7. The proportions presented in Table 3 are not clear to me. I believe this is described on page 3 lines 71-74 in the methods section. However, it would be useful if the authors provided an example of the calculation (or the written algorithm). Additionally, is this a validated approach or one developed by the authors?

🡪 We thank the reviewer for this suggestion. We fully agree that the explanation how the cumulative AB score was calculated becomes clearer if it is illustrated by an example. Therefore, we have now inserted an example in the methods section. Furthermore, we now mention that our calculations follow the approach proposed by Campbell et al. and we explain that Campbell et al. additionally calculated a mean score per person whereas we focused on the total score and did not further transfer it into a mean (page 3, lines 91-99)

*Page 3, lines 91-99: In order to describe the cumulative AB of each person during the observation period, we first multiplied the AB score of each MAC dispensed to the person during the observation period or overlapping the observation period with the length of supply (based on DDD) and then summed up the score points of all dispensations. Subsequently, these AB scores were summed up per person to calculate the cumulative AB. For example, a person receiving 200 DDDs of metformin (ACB score 1) and 30 DDDs of tramadol (ACB score 2) during the observation period had a cumulative AB of 260 (i.e., the result of 200 x 1 + 30 x 2). This method was proposed by Campbell et al. [1]. Campbell et al. further divided the cumulative AB by the number of days in the exposure period to transfer the total AB score into a mean score per person but this additional transformation was not relevant in the context of our study [1]..*

Specific Comments
(Note the line numbers changed throughout the document, making it challenging to identify exact location of comments)

🡪 We thank the reviewer for pointing out this mistake. The line numbers were changed to a continuous enumeration

1. Page 2 Line 43 (Methods): What do OPS and EBM stand for? I could not find these acronyms defined previously.

🡪We thank the reviewer for this remark. OPS stands for “Operationen- und Prozedurenschlüssel” which roughly translates to “Operations and procedure classification” and EBM-“Einheitlicher Bewertungsmaßstab” translates to “Uniform Value Scale”. We have chosen to remove the acronyms OPS and EBM from the text, as they do not provide any additional information to international readers (page 2, lines 64-66).

*Page 2, lines 64-66: Morbidities were assessed any time prior to observation period (starting from 2004) through records of ≥1 ICD-10-GM inpatient or outpatient diagnoses or records of ≥1 codes of relevant operations, procedures or outpatient services as well as participation in disease management plans.*

2. Page 1 Line 1 (Results): For consistency with the following sentence, I would recommend changing “prevalences decreased with decreasing ACB score” to “prevalence increased with increasing ACB score”. Please also specify that these are prior morbidities and medications.

🡪 We thank the reviewer for this suggestion. We have followed it and changed the sentence accordingly (page 7 lines 134-135).

*Page 7 lines 134-135: For all morbidities and medications assessed prior to start of observation period, prevalences increased with increasing ACB score (S1 Table).*

3. Page 1 line 10 (Discussion): Previous the acronym “AB” was used for “anticholinergic burden”, however, here it is spelled out. This should likely be switched to “AB”.

🡪 We thank the reviewer for pointing this out. We have abbreviated Anticholinergic burden to AB in the discussion

4. Page 1 line 19 (discussion): I believe there should be a comma after “interestingly”.

🡪 We thank the reviewer for spotting this mistake, we have corrected it accordingly.

Reviewer #2:

Thank you for the opportunity to review this interesting manuscript; which describes the assessment of anticholinergic burden among a large sample of Germans. The data source and sample are well described.

My main concern is with the measure of exposure - which is essentially supposed to be cumulative anticholinergic burden.
o Why limit to just one year of follow-up? There is a risk of underascertainment of exposure, particularly for anticholinergic medications dispensed just prior to the start of 2016.

🡪 We apologize for the fact that we have obviously created a misunderstanding regarding our study design. We think the word follow-up was misleading and we now specify in the methods section that this was a cross-sectional study aimed to describe the prevalence of anticholinergic burden in Germany stratified by age and sex group in 2016. In addition, we have now added a graphical depiction of the study design to avoid this misunderstanding (new Figure 1). We have taken different approaches to describe the prevalence of AB because we think this provides a more comprehensive picture of the situation. On the one hand, we described the persons regarding the highest category of AB reached during the study period (i.e. in 2016). In addition, we calculated a measure that allowed us to assess the proportion of AB attributable to a certain class of MAC (e.g., antidepressants) or physician specialty. We called this measure “cumulative burden” because it takes into account all dispensations in the study period (i.e. in 2016).

Regarding medication that was dispensed before the start of the study period, we included all anticholinergic medication that was dispensed in 2015 and whose treatment durations overlapped into the study period into calculation of the anticholinergic burden. In order to emphasize this point we modified the paragraph describing the assessment of exposure (pages 2-3, Lines 70-73):

*Page 2-3, Line 70-73: Exposure to MAC was assessed based on outpatient prescriptions dispensed during the observation period, i.e., in 2016. Treatment durations were estimated based on DDDs. In case MAC were dispensed before 1 January 2016 and the days of supply covered by this dispensation overlapped with the observation period, the DDDs overlapping with the observation period were also considered.*

o As I read the manuscript, the minimum follow up was only one day; this also could contribute to bias in the measure of exposure. Provide some justification for this; also perform sensitivity analyses on limiting to individual with a reasonable amount of follow up.

🡪 We hope our explanation above clarifies that the word follow-up was misleading here as we conducted a cross-sectional study. Our intention was to avoid inclusion criteria that resulted in a non-inclusive selection of the study population as we wanted to provide a comprehensive overview of AB in an unselected population sample. We fear that using a continuous observation period in 2016 as inclusion criterion would have led to excluding certain subgroups, e.g. those who died in 2016. It is important to note that there is little fluctuation in GePaRD. In our study 91% of included persons were observable for the whole year 2016. 98% were observable for 90 days or more. Moreover, only 3.3% of persons exited the study before the end of the study period due to end of a continuous insurance period. Therefore, we do not see the duration of follow-up as a source of bias requiring additional analyses. We now mention this important aspect in the discussion (page 15, lines 248-250).

*Page 15, lines 248-250: Moreover, the study population was fairly stable: 91% of included persons were observable for the whole year of 2016, 98% were observable for 90 days or more and only 3.3% exited the study before the end of the observation period due to end of continuous insurance..*

o When assessing cumulative anticholinergic exposure it is important to understand all of which medications are used, the dosage at which they are taken, and the patterns/overlap in which different medications are taken --> and all of these, over the long term. Aside from the already-identified issues with minimum and maximum follow up time for the cohort, dosage is inadequately addressed using the current methodology. A variety of publications exist that describe methods to estimate cumulative anticholinergic burden more precisely and these could be employed here.

🡪 As explained above we hope it is now clearer why we calculated the “cumulative anticholinergic burden” here. It was just used as a measure to describe some further aspects of the prevalence of AB. We fully agree that for a study aiming to determine risk associated with a high AB, a more precise approach would be needed to determine exposed and unexposed person-time, but this was not the aim of this study. We now address this point in the discussion (page 16, lines 256-259).

Page 16, lines 256-259: *Finally, cumulative AB was used in this study as a measure to further describe certain aspects regarding the prevalence of MAC prescribing. It should be kept in mind, however, that this measure might not be suitable for studies assessing the risk of outcomes associated with AB where a precise classification of exposed and unexposed time windows is important.*

After reviewing the methods and results, I am left with the feeling that there is room for inaccuracy -- and likely, underestimation -- in cumulative anticholinergic assessment. I think the authors could benefit from considering those other methods and also using sensitivity analyses to explore the impact on results.

With respect to outcomes, the prevalence of anticholinergic burden is interesting -- but really only as an intermediate on the pathway to the negative health outcomes that are the end result of high cumulative anticholinergic burden. The time period of the study is insufficient to truly understand the risk of these long term effects; and why such a short time period was selected was not expanded upon in the manuscript. Nonetheless, if the rationale for this study is that anticholinergic burden in young people is under-studied, demonstrating that this is important (because it is associated with negative health outcomes in young people) would be an important step. I think that whether anticholinergic exposure is an issue among young people remains to be established and this manuscript could delve more in to that

🡪 Thank you for this comment. As the reviewer states, the aim of this study was indeed to provide a comprehensive overview of the prevalence of AB by age and sex in an unselected population sample, including younger age groups which were understudied so far. We fully agree that risk studies would be needed in a next step to find out whether anticholinergic exposure is an issue among young people as our study only determined the prevalence of AB in this population. We now expand on this on page 14, Line 222-223

*Page 14, Line 222-223: This demonstrates the need to conduct studies on potentially harmful effects not only in older adults but also in children, adolescents, and the entire adult population.*

Minor points
-The manuscript could benefit from some proofreading/English language editing.

🡪 We thank the reviewer for this remark. The updated manuscript was proofread by a native speaker

-The discussion is wordy and could be streamlined; particularly the paragraphs that repeat the results in comparing to the published literature.

🡪 We thank the reviewer for this suggestion. We have shortened the discussion section accordingly.

6. PLOS authors have the option to publish the peer review history of their article ([what does this mean?](https://journals.plos.org/plosone/s/editorial-and-peer-review-process#loc-peer-review-history)). If published, this will include your full peer review and any attached files.


If you choose “no”, your identity will remain anonymous but your review may still be made public.


**Do you want your identity to be public for this peer review?** For information about this choice, including consent withdrawal, please see our [Privacy Policy](https://www.plos.org/privacy-policy).

Reviewer #1: No

Reviewer #2: No

[NOTE: If reviewer comments were submitted as an attachment file, they will be attached to this email and accessible via the submission site. Please log into your account, locate the manuscript record, and check for the action link "View Attachments". If this link does not appear, there are no attachment files.]

While revising your submission, please upload your figure files to the Preflight Analysis and Conversion Engine (PACE) digital diagnostic tool, <https://pacev2.apexcovantage.com/>. PACE helps ensure that figures meet PLOS requirements. To use PACE, you must first register as a user. Registration is free. Then, login and navigate to the UPLOAD tab, where you will find detailed instructions on how to use the tool. If you encounter any issues or have any questions when using PACE, please email PLOS at [figures@plos.org](mailto:figures@plos.org). Please note that Supporting Information files do not need this step.

References

1.	Campbell NL, Perkins AJ, Bradt P, Perk S, Wielage RC, Boustani MA, et al. Association of Anticholinergic Burden with Cognitive Impairment and Health Care Utilization Among a Diverse Ambulatory Older Adult Population. Pharmacotherapy: The Journal of Human Pharmacology and Drug Therapy. 2016;36(11):1123-31. doi: <https://doi.org/10.1002/phar.1843>.
