# Supplementary material for: Anticholinergic burden: First comprehensive analysis using claims data shows large variation by age and sex
Source: PLoS One. 2021 Jun 30;16(6):e0253336. doi: 10.1371/journal.pone.0253336 (PMC8244868; doi:10.1371/journal.pone.0253336)
Supplement: Attachment — Submitted filename: Reinold_et_al_2021_Answers_to_the_Reviewers.docx [file pone.0253336.s003.docx]

Journal Requirements:

Please review your reference list to ensure that it is complete and correct. If you have cited papers that have been retracted, please include the rationale for doing so in the manuscript text, or remove these references and replace them with relevant current references. Any changes to the reference list should be mentioned in the rebuttal letter that accompanies your revised manuscript. If you need to cite a retracted article, indicate the article’s retracted status in the References list and also include a citation and full reference for the retraction notice.

>> We thank the editor for this comment. We have checked and updated the reference list accordingly. We fixed a mistake where the reference

Campbell NL, Perkins AJ, Bradt P, Perk S, Wielage RC, Boustani MA, et al. Association of Anticholinergic Burden with Cognitive Impairment and Health Care Utilization Among a Diverse Ambulatory Older Adult Population. Pharmacotherapy. 2016;36(11):1123-31. Epub 2016/10/07. doi: 10.1002/phar.1843. PubMed PMID: 27711982; PubMed Central PMCID: PMCPMC5362375.

appeared in the reference list twice. This is also documented in the rebuttal letter. We did not cite any retracted manuscripts.

[Note: HTML markup is below. Please do not edit.]

Reviewers' comments:

Reviewer's Responses to Questions

**Comments to the Author**

1. If the authors have adequately addressed your comments raised in a previous round of review and you feel that this manuscript is now acceptable for publication, you may indicate that here to bypass the “Comments to the Author” section, enter your conflict of interest statement in the “Confidential to Editor” section, and submit your "Accept" recommendation.

Reviewer #1: All comments have been addressed

Reviewer #2: (No Response)

2. Is the manuscript technically sound, and do the data support the conclusions?

The manuscript must describe a technically sound piece of scientific research with data that supports the conclusions. Experiments must have been conducted rigorously, with appropriate controls, replication, and sample sizes. The conclusions must be drawn appropriately based on the data presented.

Reviewer #1: Yes

Reviewer #2: Yes

3. Has the statistical analysis been performed appropriately and rigorously?

Reviewer #1: Yes

Reviewer #2: Yes

4. Have the authors made all data underlying the findings in their manuscript fully available?

The [PLOS Data policy](http://www.plosone.org/static/policies.action#sharing) requires authors to make all data underlying the findings described in their manuscript fully available without restriction, with rare exception (please refer to the Data Availability Statement in the manuscript PDF file). The data should be provided as part of the manuscript or its supporting information, or deposited to a public repository. For example, in addition to summary statistics, the data points behind means, medians and variance measures should be available. If there are restrictions on publicly sharing data—e.g. participant privacy or use of data from a third party—those must be specified.

Reviewer #1: Yes

Reviewer #2: Yes

5. Is the manuscript presented in an intelligible fashion and written in standard English?

PLOS ONE does not copyedit accepted manuscripts, so the language in submitted articles must be clear, correct, and unambiguous. Any typographical or grammatical errors should be corrected at revision, so please note any specific errors here.

Reviewer #1: Yes

Reviewer #2: Yes

6. Review Comments to the Author

Please use the space provided to explain your answers to the questions above. You may also include additional comments for the author, including concerns about dual publication, research ethics, or publication ethics. (Please upload your review as an attachment if it exceeds 20,000 characters)

Reviewer #1: Thank you for addressing the majority of the comments raised during the initial review. I believe the authors have substantially improved the manuscript. However, I do have a couple of additional questions that I would appreciate if the authors could address further regarding the study design.

1. I do appreciate that the authors have tried to generate a figure for the study design, however, it is not quite clarifying the cross sectional design to me. Particularly regarding the follow-up and dates that the morbidities and medications were identified on. For example, if the inclusion was met on February 1st 2016 for a patient, I assume the morbidities were assessed from January 1st 2004 through to January 31st 2016 (one day before the inclusion date), and medications were identified from January 1st 2015 through to January 31st 2016 (again one day before inclusion)? This is the level of detail in the figure that would be helpful to better understand the design.

>> The reviewer’s understanding of the time frames is correct. We have heeded the reviewer’s suggestion and created a new version of figure 1 where we clarified the timeframes in which morbidities and medications were assessed. In particular, we have now defined the day of inclusion in a footnote so that it is clear to which date the periods refer.

2. On the point of morbidity and medication assessment prior to inclusion, this information (e.g., what were to morbidities and medications) is not summarized in the paper? I imagine this information could be helpful to understand differences in the patients with AB. Therefore, I wonder why this is excluded from the results since it appears to be assessed?

>> We thank the reviewer for this comment; In addition to the detailed information provided in Table S1, we have now added the following paragraph to summarize information on morbidity, medication as well as healthcare utilization in the study population, stratified by ACB status (ACB=1, ACB=2, ACB≥3 and persons without ACB) on page 7 lines 141-146

Page 7 lines 141-146: For example, compared to persons with lower or no ACB, persons with ACB≥3, had higher prevalences of psychiatric and behavioral, musculoskeletal as well as endocrine and metabolic diseases. They were prescribed medications from a higher number of different prescribers and had higher prevalences of cardiovascular therapy, analgesics and psychiatric medication. Moreover, persons with ACB≥3 were, on average, more frequently hospitalized, remained hospitalized for longer periods and had a higher prevalence of nursing home residency and obesity.

2. I am still unsure if this is truly a cross-sectional design as it does not appear to be a single snapshot of a patient at a given time-point. But instead there appears to be follow-up. For example, in the methods section and Figure 1, it states that patients were "followed until (i) death, (ii) start of hospitalisation in 2016 with a duration of >=90-days, (iii) end of continuous insurance period, or (iv) end of observation period, whichever occurred first". Based on this statement, the design still reads like a cohort study as you are following individual patients over time. With this in mind I wonder if it is a descriptive cohort study rather than a true cross-sectional design? Can the authors further clarify this. I think it would also help address the concerns from both reviewers regarding the potential for misclassification on the AB.

I understand the authors aim of the paper is only to assess the prevalence of AB in a younger population, which is of high interest. And while a cohort study that compares outcomes is not the aim, I do think further clarification on the follow-up and time of measurements is still warranted so that future work can replicate these findings and potentially conduct a cohort study to assess health outcomes.

>> We apologize that some parts of the description of the study design still created confusion. We fully understand that the expression “followed until…” was misleading here. We have now re-written this part of the methods section and hope it is clear now that this sentence should simply describe the observation period in 2016 that was used to assess the use of MAC. In a cross-sectional study based on primary data, one would ask patients, for example, about their MAC use in the past year. An assessment period of one year for MAC use seems reasonable to ensure that the assessment of MAC is robust regarding potential seasonal variations. In our study which was based on secondary data, this corresponds to defining an assessment period for MAC use of one year. Maybe this comparison is also helpful to explain the other measurement periods. In a primary data study, one would ask patients, for example, if they ever had a certain comorbidity (e.g. cancer). In our study based on secondary data, it is not possible to ask patients directly and pose the “ever” question. To compensate for that as much as possible, we used all the information available on this person in the database before 2016. This may also explain the measurement period we used for co-medication. In a primary data study, one would ask the patient about recent medication use (e.g. past 12 months). Analogously, we used an assessment period of one year to assess co-medication. We now expand on this also in the methods section and hope that this clarifies the study design.

Page 2 lines 60-62: For all included persons, the available (continuous) observation period in 2016 was used to assess the use of MAC. For persons with a hospitalization starting in 2016 and with a duration of ≥90 days, MAC use was only assessed until the start of this hospitalization (Figure 1).

Page 2 lines 65-71: The coding of morbidities was assessed any time prior to observation period (starting from 2004) through records of ≥1 ICD-10-GM inpatient or outpatient diagnoses or records of ≥1 codes of relevant operations, procedures or outpatient services as well as participation in disease management plans. This approach, i.e. taking into account all information on morbidity available for a person before 2016, aims to compensate for the fact that with secondary data, a person cannot be asked if he or she ever had a certain disease, as it would be done in a study based on primary data. Treatment with medication excluding MAC was assessed within 365 days before start of observation period (excluding start of observation period) based on records of ≥1 outpatient dispensations.

>> We also would like to point out that other cross-sectional studies based on claims data have used a similar approach in regards to the definition of observation periods, so for claims data analysis this is not an unusual approach (e.g. Simon et al, Pediatr Rheumatol Online J. 2020; 18: 43. (<https://www.ncbi.nlm.nih.gov/pmc/articles/PMC7275412/>) or Mapel et al BMC Health Serv Res. 2011; 11: 43. (<https://www.ncbi.nlm.nih.gov/pmc/articles/PMC3050697/>)

Reviewer #2: Thank you for the revisions. I think the study design is much clearer now and the explanatory figures included helpful.

The work done to explain that its really a cross-sectional prevalence (assessed over a one year period) is clarifying; but at the same time, I feel the implications of this requires some discussion or at least some further expansion in the limitations.

To the best of my knowledge (but this would be worth the authors discussing in any case) the time period over which the cumulative effects of anticholinergic burden accrue are unclear. The important point about high anticholinergic burden -- as the authors are well aware -- is not that people are on a number of medications, but rather than these medications together create an exposure that results in negative health outcomes like falls and dementia. But over what time period does this exposure need to occur? Does it matter if someone has a high level of anticholinergic burden for a 30 day period while a set of medications interact? Or does that cumulative exposure need to be over a 30 day period or 60 day period or for >1 year? How does the study design chosen illuminate the relationship between exposure and outcomes; or in another way, do we know that the exposures measured and reported here are clinically relevant?

To be clear, I don't think the authors need to actually answer all the questions above (and indeed, I believe some are presently unanswerable); but rather this is the context in which to discuss the strengths and limitations of their study design. I am unclear why reporting the results of a cross sectional measure of 'cumulative' (as the authors define is) is an important finding -- but i would welcome the authors defense of why this is a valid and important measure, through expansion of the discussion section.

>> We fully agree that the points the reviewer raised are very important and - even though partly unanswerable - should be mentioned in the discussion to sensitize the reader to uncertainties in the field. Therefore, we now expand on this in the discussion in a separate paragraph (page 15, lines 244-253). With respect to the ‘cumulative” AB: We hope our answer regarding the study design we provided in response to reviewer 1 explains to a certain extent why we think this measure is not contradictory to a cross-sectional study. Also in a cross-sectional study based on primary data, one would ask a patients about MAC use e.g. in the past year, and then there are different options how to categorize / classify this information to describe the prevalence of AB. Apart from describing the persons regarding the highest category of AB reached during the study period, we calculated this additional measure because – unlike the aforementioned measure - it allowed us to assess the proportion of AB attributable to a certain class of MAC or to a certain physician specialty. We think this additional measure is helpful as it provides insights into the medication and sectors of care that contribute to the AB in the population.

Page 15 lines 244-253: Our study showed that there are persons with an AB considered to be clinically relevant in all age groups. This demonstrates the need to conduct studies on potentially harmful effects not only in older adults but also in children, adolescents, and the entire adult population. However, it has to be kept in mind that there are a lot of unanswered questions in regards to how AB can cause or contribute to clinically relevant adverse effects. For example, the time period over which the cumulative effects of anticholinergic burden may accrue and possibly produce harms are unclear. Also the role of type and dosage of single MACs and their overlap are not well understood. When planning a study on the risk of AB, this means that classifying persons as exposed or unexposed bears a high level of uncertainty, so robustness of findings would need to be assessed by comprehensive sensitivity analyses. Also in many other regards, studies on the risk of outcomes associated with AB are challenging, e.g. regarding issues such as confounding by indication, unmeasured confounding and time-varying exposure.

At the very least, I would think a clear limitation to the design should be added to state that longitudinal assessment of anticholinergic burden that accounts for dose is what would be needed to understand the link between anticholinergic burden exposure and negative health outcomes -- particularly in the younger population who may have higher than expected anticholinergic burden but to my knowledge have never been shown to develop the negative health outcomes observed in older adults.

I recognize that the authors have inserted this limitation related to the above: 'It should be kept in mind, however, that this measure might not be suitable for studies assessing the risk of outcomes associated with AB where a precise classification of exposed and unexposed time windows is important.' However in my mind, that is the important goal of this type of work. So discussing/addressing that limitation more fulsomely would be useful.

>> We apologize that we had not sufficiently addressed this limitation yet. We now expand on this in the limitations section and – as also mentioned above –have added a paragraph addressing the open questions in this regard.

Page 16 lines 274-279: Nonetheless, this approach is not equivalent to other studies that had more information on dosage and used more sophisticated methods to take it into account. Finally, in our study we have not assessed AB in a longitudinal manner, which – in view of the aforementioned unanswered questions about clinically relevant AB levels – would be essential in a subsequent risk study to understand the potential link between AB exposure and negative health outcomes. Such risk studies are particularly needed in the younger population where it is even less clear if such a link exists at all.

7. PLOS authors have the option to publish the peer review history of their article ([what does this mean?](https://journals.plos.org/plosone/s/editorial-and-peer-review-process#loc-peer-review-history)). If published, this will include your full peer review and any attached files.


If you choose “no”, your identity will remain anonymous but your review may still be made public.


**Do you want your identity to be public for this peer review?** For information about this choice, including consent withdrawal, please see our [Privacy Policy](https://www.plos.org/privacy-policy).

Reviewer #1: No

Reviewer #2: No
